# 311. Impact of Non-alcoholic Fatty Liver Disease on Clinical Outcomes in Patients with COVID-19

**DOI:** 10.1093/ofid/ofab466.513

**Published:** 2021-12-04

**Authors:** Nina Vrsaljko, Lara Samadan, Jelena Budimir, Mirjana Balen Topic, Ivan Kurelac, Adriana Vince, Neven Papić

**Affiliations:** 1 University Hospital for Infectious Diseases Zagreb, Zagreb, Grad Zagreb, Croatia; 2 School of Medicine, University of Zagreb, Croatia, Zagreb, Grad Zagreb, Croatia

## Abstract

**Background:**

Non-alcoholic fatty liver disease (NAFLD) is the most common liver disease with a prevalence up to 30%. NAFLD is strongly associated with components of metabolic syndrome, already recognized as risk factors for worse outcomes in COVID-19. However, the impact of NAFLD on COVID-19 is not well characterized. The aim of this study was to investigate a possible association between NAFLD and COVID-19 severity and outcomes.

**Methods:**

A prospective observational study included consecutively hospitalized adult patients with severe COVID-19 at the University Hospital for Infectious Diseases in Zagreb, Croatia between March and June 2021. On admission patients were screened for fatty liver by the ultrasound and subsequently diagnosed with NAFLD according to current guidelines. Demographic, clinical and laboratory data was collected and correlated to clinical outcomes.

**Results:**

Of the 112 patients included in the study, 77 (68.7%) had NAFLD (59.7% males; median age of 62, IQR 54-66 years). Except for higher prevalence of obesity in NAFLD group (61.0% vs 17.1%) there were no differences in other comorbidities. NAFLD group had higher inflammatory markers CRP (96, IQR 51-138 vs 59, IQR 29-99mg/L) and IL-6 (129, IQR 44-169 vs 25, IQR 8-56pg/mL). Steatosis stage showed positive correlation with BMI, waist/hip ratio, CRP, PCT, IL-6, AST, ALT, LDH and fibrinogen. Steatosis stage correlated with clinical status at the 7-category scale on admission and at days 7, 14 and 28. Patients with NAFLD had longer duration of hospitalization (9, IQR 6-15 vs 6, IQR 5-11 days, p=0.024), more frequently required noninvasive ventilation or high-flow oxygen (24.7% vs 5.7%, p=0.018) and had higher rate of pulmonary embolism (22.1% vs 5.7%, p=0.024). There was no difference in mortality. The median value for clinical status on the ordinal scale at day 7 was significantly higher in NAFLD group at days 7 and 14, as presented in Fig. 1. Multivariable analysis identified age > 65 (OR 3.6, 95%CI 1.3-10.9), LDH > 350 (OR 8.1, 95%CI 2.7-29.4), NAFLD (OR 3.9, 1.1-20.5) and pulmonary embolism (OR 10.4, 2.7-48.3) associated with adverse outcomes at day 28.

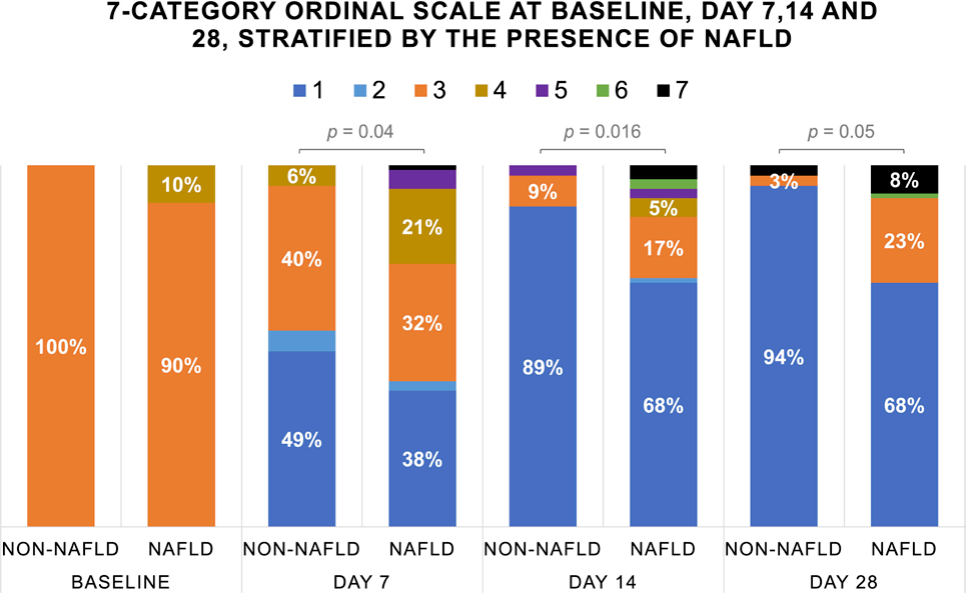

The figure shows the patients’ clinical status as assessed on the seven-category ordinal scale on admission and at day 7, 14 and 28, according to the presence of NAFLD. Categories on the ordinal scale were as follows: 1, discharged or ready for discharge; 2, hospitalization in a non–intensive care unit (ICU) without supplemental oxygen; 3, non–ICU hospitalization with supplemental oxygen; 4, ICU or non–ICU hospitalization with noninvasive ventilation or high-flow oxygen; 5, ICU hospitalization with mechanical ventilation; 6, ICU hospitalization with extracorporeal membrane oxygenation or mechanical ventilation and additional organ support; and 7, death.

**Conclusion:**

Our data suggests that NAFLD is associated with COVID-19 severity and might be linked to adverse outcomes in hospitalized patients.

**Disclosures:**

**All Authors**: No reported disclosures

